# An exploratory study of social media users’ engagement with COVID-19 vaccine-related content

**DOI:** 10.12688/f1000research.51210.2

**Published:** 2021-06-16

**Authors:** Md. Sayeed Al-Zaman

**Affiliations:** 1 Department of Journalism and Media Studies, Jahangirnagar University, Dhaka, 1342, Bangladesh; 2 Department of Media and Technology Studies, University of Alberta, Edmonton, Alberta, T6G 2E6, Canada

**Keywords:** COVID-19 vaccine; Facebook; social media content; user engagement; medicine.

## Abstract

***Background:*** Facebook, as the world’s most popular social media platform, has been playing various important roles throughout the COVID-19 pandemic, allowing users to produce and share health-related information that both eases and complicates public health communication. However, the characteristics of vaccine-related Facebook content and users’ reaction to the vaccine issue has been an unexplored area to date.

***Methods:*** To fill the previous knowledge-gap, this exploratory study wants to understand the communication climate of Facebook on the COVID-19 vaccine issue, including the nature of dominant content and users’ engagement patterns with them. Therefore, the study analyzes the 10,000 most popular Facebook posts with the highest interactions on the vaccine issue.

***Results:*** The results show that Facebook users prioritize more vaccine-related news links (71.22%) over other content. The declining interactions on the issue suggests that interaction growth mainly depends on positive news on the vaccine. Finally, users’ reaction to the vaccine issue is dominantly positive, though they may show a highly negative attitude toward vaccine misinformation.

***Conclusions:*** A few limitations and strengths of this study are discussed along with values and implications. This study for the first time analyzes Bangla language-based Facebook content related to the COVID-19 vaccine issue, which is largely overlooked in global academic research.

## Introduction

This exploratory study seeks to understand the characteristics of coronavirus disease 2019 (COVID-19) vaccine-related social media content and how users engage with them. COVID-19 has already claimed 1.76 million lives worldwide, making the public health condition critical. Meanwhile, lockdown and social distancing interrupt face-to-face human communication ( Limaye *et al.*, 2020). Such a situation lets social media bridge the communication gap by delivering necessary information ( Goel & Gupta, 2020; González-Padilla & Tortolero-Blanco, 2020). Researchers categorized COVID-19 online information into 11 types: valid information, comforting information, perplexing information, misinformation, disinformation, shocking information, contradictory information, doubtful information, progressive information, postponed information, and confidential information ( Ashrafi-Rizi & Kazempour, 2020). Two broader categories may represent all of them: useful information that helps public health communication, and faulty information that interrupts public health communication. For example, social media was responsible for fake COVID-19 prescriptions and medications that claimed many human lives ( Islam *et al.*, 2020). On the contrary, infographics containing COVID-19 health information delivered through Twitter and WeChat helped rapid COVID-19 knowledge dissemination ( Chan *et al.*, 2020). Similarly, on the one hand, social media helps to run positive and effective virtual campaigns for the COVID-19 vaccine around the world, and, on the other hand, vaccine opposition and misinformation obstruct the vaccination process ( Bonnevie *et al.*, 2020). A report says 31 million Facebook users follow anti-vaccine groups with 17 million YouTube users subscribing to similar accounts ( Burki, 2020). Therefore, it is imperative to understand social media users’ sentiments regarding the COVID-19 vaccine, and the nature of vaccine-related content that dominates social media platforms.

Previous studies from both the public health and communication domain dealt with different aspects of the COVID-19 vaccine, such as vaccine hesitancy ( Bonnevie *et al.*, 2020; Jamison *et al.*, 2020; Puri *et al.*, 2020), vaccine misinformation ( Jamison *et al.*, 2020; Loomba *et al.*, 2020), media, and the COVID-19 vaccine ( Olijo, 2020). However, no attention has been paid to social media users’ engagement with the COVID-19 vaccine issue. The present research attempts to fill this gap.

## Methods

### Study objectives

This research attempts to understand the communication climate of Facebook surrounding the COVID-19 vaccine issue, the nature of dominant content and users’ engagement patterns with them.

### Data source & search strategy

Facebook is the most popular social media platform in Bangladesh (94.46%), compared to YouTube (3.31%) and Twitter (0.3%), so we selected it as the source of data for this study. we collected the data from Facebook pages using CrowdTangle, a public insights tool owned and operated by Facebook. It can access public pages and groups to collect various types of data. However, we excluded group data in this research because Facebook groups and pages are different in terms of users’ interaction patterns, participation rates, and security issues. To collect the data from relevant Facebook posts, we conducted a keyword search. Most of the Facebook users in Bangladesh who write in the Bangla language use the Bangla alphabet. For that reason, we determined the following Bangla keywords: “corona vaccine,” “coronar vaccine,” “corona tika,” “coronar tika,” “corona oshudh,” “coronar oshudh,” “corona protishedhok,” and “coronar protishedhok.” All search terms can be translated as *corona vaccine* or *vaccine of the corona*.

### Data collection & analysis

I set the timeline from 8 March to 2 December 2020. The first case of coronavirus in Bangladesh was identified on 8 March 2020. As Facebook is an interactive platform, so we collected the data on 17 December 2020 (15 days after the end date of the data collection period) after stabilizing the threads. The search result produced 37,460 Facebook posts relevant to the topic. Of them, we selected the top 10,000 posts for the final analysis based on their total interactions, which was 26.70% of the total search results: These posts were from 1,087 different Facebook pages, on average 9.20 posts from each page. We categorized the content into six types: link, live video, native video, photo, status, and YouTube video. In Facebook, each of the mentioned content can usually have the following definitions and modes: a link and a photo can be shared with or without any text; live video can be completed or scheduled; native video is video that is uploaded directly to Facebook; a status is mainly text-based, often contains other elements, such as tags; YouTube videos are often embedded/shared on Facebook. We measured the users’ engagement based on nine interaction parameters, including six Facebook reactions: comments (mean [M]=32.90, standard deviation [SD]=140.06), shares (M=145.17, SD=1345.86), like (M=1377.04, SD=4792.64), love (M=74.98, SD=788.35), wow (M=6.50, SD=40.33), haha (M=52.79, SD=408.28), sad (M=16, SD=372.76), angry (M=3.67, SD=55.05), and care (M=5.40, SD=51.67). In this study, we followed a bivariate statistical analysis and a simple time-series analysis with one-day intervals. The numbers and percentages are presented in tables, time-series data are presented in graphs.

### Framing & interpreting Facebook reactions

In February 2016, Facebook introduced five basic reactions: love, sad, wow, haha, and angry, alongside like or thumbs-up. These reactions were influenced by the six universal reactions: fear, anger, joy, sadness, disgust, and surprise ( Gu *et al.*, 2019), proposed by Ekman (1992) based on human facial expressions ( Keltner *et al.*, 2019). However, studies found that disgust and anger, and fear and surprise share similar characteristics, meaning the four following emotions should be considered as the actual basic emotions: anger, joy, sadness, and surprise ( Jack *et al*., 2014). Unlike basic emotions, Facebook reactions represent more varied and often contradictory expressions: *like* or *thumbs-up* usually expresses likeness, agreement, happiness, approval, and acceptance; *love* expresses positivity, affection, empathy, support, kindness, and liking something with feeling; *sad* expresses empathy, grief, tragedy, and support; *haha* expresses laughter, fun, mockery, and often rejection; *wow* expresses muted disdain, surprise, immediate shock, and skepticism; and *angry* expresses denial, aggressiveness, strong and overt disdain, and dislike ( Al-Rawi, 2020).

 In contrast to the idea of basic emotions, the circumplex model of affect proposes that human emotion is more complex and diverse than the basic emotion theory explains ( Russell, 1980). At least 28 interconnected emotional states of humans can be defined with two variables: emotional valence or sentiment and emotional arousal or intensity ( Citron *et al.*, 2014; Freeman *et al*., 2020; Yang & Chen, 2012). This two-dimensional model of human emotions defines emotional valence as positive and negative and emotional arousal as high and low ( Preoţiuc-Pietro *et al.*, 2016). In this study, we tried to position Facebook reactions within this paradigm but with little modification. For valence, we defined like, love, wow, and care as positive, and angry, sad, and haha reactions as negative valence ( Giuntini *et al.*, 2019). As the number of Facebook emotions is limited, unlike the circumplex model that has 28 distinct but relative emotions, we defined the seven reactions as follows: love, care, angry, and wow as high arousal, and like and sad as low arousal. That means positive high includes love, care, and haha; positive low includes like; negative high includes angry and wow; negative low includes sad. Although *like*, according to its defined meanings, belong to the lower arousal dimension, its degree is moderate, which is closer to the high arousal dimension (see Russell, 1980). Again, Al-Rawi (2020) defined *wow* as positive, we defined it as negative based on the findings of Jack *et al*. (2014) (i.e., fear and surprise are the same, and *wow* expresses surprise or shock, which is negative). It is important to note that expressions through these reactions vary widely, and a reaction may express contradictory emotions: haha both expresses joy and refutation. It makes this reaction’s position in the paradigm uncertain: it can be positive high and negative high. For such ambiguous nature, previous studies excluded haha from their interpretations (e.g., Al-Rawi, 2020).

## Results

### Analysis of content

**Figure 1.**
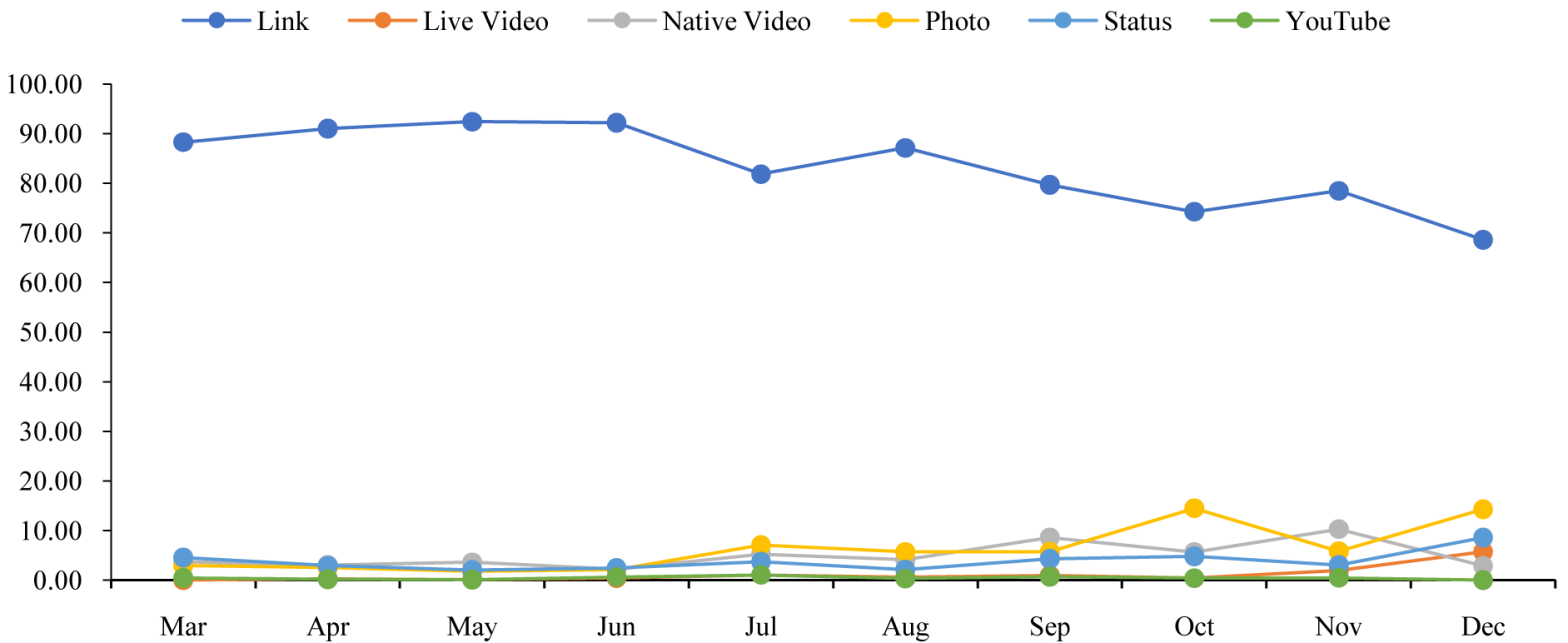
Content distributions during the time.

Of the six content types, link ( *n=*8,614; 86.14%) was dominant, followed by photos ( *n=*491; 4.91%) and native videos ( *n=*466; 4.66%); YouTube video ( *n=*52; 0.52%) was on the bottom of the list ( Table 1). During the time span, links were shared the most in July ( *n=*1,696; 19.69%), followed by August ( *n=*1,331; 15.45%); photos were shared the most in July ( *n=*147; 29.94%) as well, followed by October ( *n=*72; 14.66%). July (20.73%) had the highest percentage of content, followed by August (15.28%) and April (14.33%). Figure 1 shows that the percentage of the links content type was dropping gradually with a few fluctuations. On the other hand, the percentages of live video and status were slowly increasing with a few drops. However, except for the line of links, other lines do not show any significant changes.

**Table 1.** Content types and their percentages.

Months	Total contents ( *n*, %)	Content types ( *n*, %)					
		Link	Live Video	Native Video	Photo	Status	YouTube
Mar	639 (6.39)	564 (6.55)	0 (0.00)	24 (5.15)	19 (3.87)	29 (9.27)	3 (5.77)
Apr	1433 (14.33)	1304 (15.14)	4 (6.25)	44 (9.44)	36 (7.33)	42 (13.42)	3 (5.77)
May	1223 (12.23)	1130 (13.12)	1(1.56)	44 (9.44)	22 (4.48)	25 (7.99)	1 (1.92)
Jun	1231 (12.31)	1135 (13.18)	5 (7.81)	27 (5.79)	26 (5.30)	30 (9.58)	8 (15.38)
Jul	2073 (20.73)	1696 (19.69)	22 (34.38)	109 (23.39)	147 (29.94)	77 (24.60)	22 (42.31)
Aug	1528 (15.28)	1331 (15.45)	9 (14.06)	63 (13.52)	87 (17.72)	33 (10.54)	5 (9.62)
Sep	719 (7.19)	573 (6.65)	7 (10.94)	62 (13.30)	41 (8.35)	31 (9.90)	5 (9.62)
Oct	497 (4.97)	369 (4.28)	2 (3.13)	28 (6.01)	72 (14.66)	24 (7.67)	2 (3.85)
Nov	622 (6.22)	488 (5.67)	12 (18.75)	64 (13.73)	36 (7.33)	19 (6.07)	3 (5.77)
Dec	35 (0.35)	24 (0.28)	2 (3.13)	1 (0.21)	5 (1.02)	3 (0.96)	0 (0.00)
Total	10000	8614 (86.14)	64 (0.64)	466 (4.66)	491 (4.91)	313 (3.13)	52 (0.52)

### Interaction analysis

**Figure 2.**
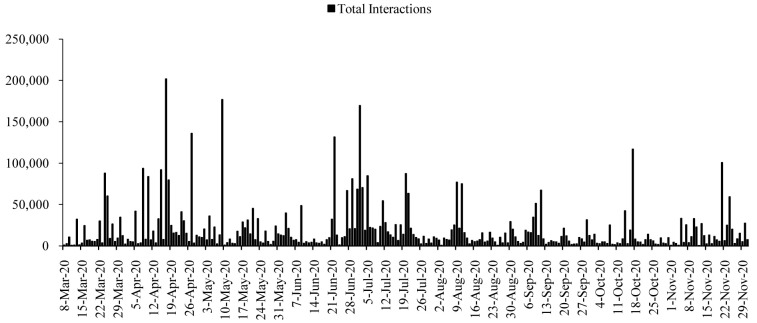
Interaction distributions during the time.

All posts generated 17,144,403 interactions during the time span. The numbers of interactions were higher in the first half of the period, roughly from April to July 2020 ( Figure 2). The interactions reached to the highest on 17 April 2020 ( *n=*201,834), with a few more surges on 27 April ( *n=*135,941), 9 May ( *n=*176,936), 22 June ( *n=*131,564), and 2 July ( *n=*169,524). The average interactions dropped in the following months, and again increased slightly in the mid-October 2020 and late-November 2020. However, except 17 October ( *n=*116,743) and 21 November ( *n=*100,619), the interactions were dropping gradually after July 2020 till December 2020.

Of all content types, links (71.22%) received the highest interactions, followed by native videos (16.52%), while YouTube videos (0.54%) received the lowest interactions. In all content types, *like* as a reaction had the highest percentages: in all except status, the second-highest was love; in status (11.16%), the second-highest was haha ( Table 2). A comparison of interaction percentages among the content types shows that YouTube video (4.16% and 1.35%) received relatively higher percentages of comments and care in its interaction types, followed by native videos (4.05%); native videos (19%) were shared more often, followed by live video (8.33%). Of the six reactions, links received relatively higher percentages of likes (84.14%); photos received higher percentages of love (16.80%); statuses received higher percentages of wow (0.65%), haha (11.16%), and sad (5.58%).

**Table 2.** Interaction numbers and their percentages within the content types.

Content types	Total interactions ( *n*, %)	Interaction numbers and percentages within content types ( *n*, %)								
		Comments	Shares	Likes	Love	Wow	Haha	Sad	Angry	Care
Link	12210941 (71.22)	233296 (1.91)	815980 (6.68)	10274354 (84.14)	358815 (2.94)	45967 (0.38)	349074 (2.86)	74221 (0.61)	31393 (0.26)	27841 (0.23)
Live Video	239176 (1.40)	9692 (4.05)	19929 (8.33)	189173 (79.09)	15924 (6.66)	786 (0.33)	1659 (0.69)	600 (0.25)	311 (0.13)	1102 (0.46)
Native Video	2831618 (16.52)	28472 (1.01)	538104 (19)	2067440 (73.01)	128542 (4.54)	9016 (0.32)	31606 (1.12)	14841 (0.52)	2350 (0.08)	11247 (0.40)
Photo	1117323 (6.52)	36936 (3.31)	53840 (4.82)	719088 (64.36)	187739 (16.80)	4671 (0.42)	69900 (6.26)	32649 (2.92)	1762 (0.16)	10738 (0.96)
Status	652504 (3.81)	16714 (2.56)	20681 (3.17)	451965 (69.27)	47026 (7.21)	4256 (0.65)	72842 (11.16)	36441 (5.58)	799 (0.12)	1780 (0.27)
YouTube	92841 (0.54)	3866 (4.16)	3192 (3.44)	68378 (73.65)	11760 (12.67)	261 (0.28)	2845 (3.06)	1223 (1.32)	65 (0.07)	1251 (1.35)

Of the total interactions during the period, 1.92% ( *n=*328,976) were comments and 8.47% ( *n=*1,451,726) were shares ( Table 3). Of the six reactions, likes were the highest (80.32%), followed by love (4.37%) and haha (3.08%); angry (0.21%) had the lowest share. Within all interaction types, link had the highest percentages: 70.92% comments, 56.21% shares, 74.61% like, 47.85% love, 70.77% wow, 66.12% haha, 46.40% sad, 85.59% angry, and 51.60% care ( Table 3). Within comments (11.23%) and love (25.04%), photo had the second-highest percentages; within haha (13.80%) and sad (22.78%), status had the second-highest percentages; within shares (37.07%), likes (15.01%), wow (13.88%), angry (6.41%), and care (20.84%), native video had the second-highest percentages.

**Table 3.** Interaction numbers and their percentages within the interaction types.

Reactions	Total interactions ( *n*, %)	Interaction percentages within interaction types					
		Link	Live Video	Native Video	Photo	Status	YouTube
Comments	328976 (1.92)	70.92	2.95	8.65	11.23	5.08	1.18
Shares	1451726 (8.47)	56.21	1.37	37.07	3.71	1.42	0.22
Likes	13770398 (80.32)	74.61	1.37	15.01	5.22	3.28	0.50
Love	749806 (4.37)	47.85	2.12	17.14	25.04	6.27	1.57
Wow	64957 (0.38)	70.77	1.21	13.88	7.19	6.55	0.40
Haha	527926 (3.08)	66.12	0.31	5.99	13.24	13.80	0.54
Sad	159975 (0.93)	46.40	0.38	9.28	20.41	22.78	0.76
Angry	36680 (0.21)	85.59	0.85	6.41	4.80	2.18	0.18
Care	53959 (0.31)	51.60	2.04	20.84	19.90	3.30	2.32

## Discussion

### Main objectives

This study for the first time attempts to explore the nature of content and public engagement in the COVID-19 vaccine issue on Facebook. To provide some novel insights, the top 10,000 most interactive and relevant Facebook posts have been analyzed following a combination of three communication research techniques: time series analysis, network analysis, and sentiment analysis.

### Key findings

This exploratory research has a few main findings. Vaccine-related links were the highest in percentage (86.14%) of all Facebook content during the period and the highest shared content (56.21%) as well, which counters the result of Baresch *et al*. (2011) that showed Facebook users’ infrequently (49%) share news through links. Links shared in social media platforms are mostly news items primarily from different online news portals ( Baresch *et al.*, 2011), which infers that Bangladeshi Facebook users mostly consume news items related to the COVID-19 vaccine. However, the decline of links suggests that users are increasingly becoming less interested in vaccine-related news. Kleis Nielsen *et al*. (2020) also found a similar tendency that using Facebook for COVID-19 news and information is declining: from 10 April to 24 June 2020, the percentage declined from 24 to 12. Unlike the present study, however, the previous study considered all COVID-19 issues instead of *only* the vaccine issue and produced a similar result.

The interactions were higher during the first half of the period but decreased with time. Interactions surge mainly due to vaccine-related news irrespective of its valence (i.e. positive and negative). However, it seems positive news helps interactions to grow more than negative news. For example, two crucial vaccine-related positive news items from mainstream media influenced the interaction to reach its highest on 17 April: “The University of Oxford declared that the vaccine for COVID-19 would come in September,” and “The trial of COVID-19 vaccine would begin from the next week” (e.g. Daily Jugantor). The interaction reached its second-highest on 27 April due to a few more positive news events: “Corona vaccine production will start from May this year,” and “India’s Serum Institute will start producing COVID-19 vaccine soon” (e.g. Bangla Tribune, DW). Nevertheless, no significant positive follow-ups occurred in the consecutive months, so the interaction lines remained steady most of the time. Another surge on 17 October was for positive news articles: “Russia produced the first COVID-19 vaccine in the world,” and “Bangladesh’s Globe Biotech claimed the invention of COVID-19 vaccine” (e.g. BBC News, Prothom Alo).

 Like and love are the two leading reactions, meaning the valence of users’ reaction to vaccine-related content is positive and, to some extent, highly intense. On the other hand, the lowest instances of the angry reaction indicate users’ minimal negative emotion toward the issue. Both tendencies indicate a positive valence with from moderate to high arousal, suggesting Facebook users’ optimistic attitude toward the COVID-19 vaccine. But note that users’ sentiments can be different for different content types: link has more positive valence, whereas status has comparatively more negative valence. However, news links often produce discontent among users, perhaps due to presenting unsatisfactory and undesired information, such as the cancellation and failure of vaccine, and demystified fake news. In practice, the COVID-19 vaccine produced uncertainties among the public around the world, often allowing many to produce vaccine-related misinformation and fake prescriptions ( Limaye *et al.*, 2020). Also, during the pandemic period, COVID-19 misinformation is highly prevalent in social media platforms like Facebook ( Al-Zaman *et al.*, 2020; Islam *et al.*, 2020).

Another finding shows that links about the vaccine generate more interactions than others. More specifically, links are shared more and generate more comments compared to the other content types. Previous studies revealed that the primary reason for Facebook users’ news link-sharing behavior is to share important news with others that might be helpful ( Baek *et al.*, 2011). It allows easier dissemination of news and sociality, the attachment to other users. In this regard, the present study expands on the previous knowledge, adding that users not only share links containing important information but also links allow more users to engage in discussion.

## Conclusion: Limitations & strengths

This study has a few limitations. First, Facebook only introduced the care reaction in April 2020. It means interaction data before April could not include care responses, which may have impacts on the overall percentages. Second, Bangla is a complex language: diverse expressions and spellings of words can indicate the same thing. Therefore, though we searched for a single topic, our search terms were not likely to incorporate all posts. More search terms, such as “COVID-19 vaccine” and “COVID-19 medicine” could yield more search results that could have led to different findings. Third, although this study explored the content types, it failed to explain what these contents contain: vaccine news, instructions, or something else? Further research should explore what types of vaccine-related information Facebook users really consume. Fourth, previous studies found that, though links most likely contain additional text or status, they often contain photos as well ( Baresch *et al.*, 2011), often making the three content types (i.e. link, photo, and status) difficult to categorize separately. The data for this study were automated and collected using CrowdTangle, which left room for doubt regarding this issue. Finally, sentiment analysis based on only Facebook reactions and excluding textual analysis may not be representative, and, to some extent, could produce unreliable results. For example, the like reaction could be a result of the absence of any particular emotion ( Giuntini *et al.*, 2019).

Apart from these limitations, this study has strengths in a few areas. First, Bangla language-based Facebook content is largely overlooked in academic research, perhaps due to the absence of research interest or research efficiency among researchers. The present study filled this gap a little. Second, how Facebook users react to the issue of the COVID-19 vaccine is still an unexplored but necessary area of research. In this regard, the novel findings of this macro study may help psychology and communication researchers to understand the diverse reactions and emotional positions of Bangladeshi Facebook users on the COVID-19 vaccine issue. Third, this study provides new perspectives to rethink social media users’ views toward the COVID-19 vaccine. While previous studies emphasized vaccine hesitancy, negatively portrayed social media users’ sentiments toward the COVID-19 vaccine, and found that vaccine opposition was rising among social media users, the present study showed that social media users are more positive toward the vaccine issue, which the past studies could barely identify ( Bonnevie *et al.*, 2020; Puri *et al.*, 2020). Finally, this research dealt with a larger dataset than the previous studies on the COVID-19 vaccine ( Jamison *et al.*, 2020; Rovetta & Bhagavathula, 2020), which makes the results more reliable. This is an exploratory study that may not provide conclusive findings in some cases. Therefore, further research is required to provide more insights in this regard.

## Data availability

Harvard Dataverse: An exploratory study of social media users’ engagement with COVID-19 vaccine-related contents

https://doi.org/10.7910/DVN/RCWH9D ( Al-Zaman, 2021)

This project contains the following underlying data:

- COVID_medicine_posts_data.xlsx
  The data in this dataset were collected using the CrowdTangle platform. The Excel dataset includes six sheets. The first sheet titled “COVID-19 vaccine” contains the raw data with 31 different variables, such as sources (e.g. FB pages) of data, date of the posts, content types, users’ engagement indicators (e.g. number of reactions, comments, and shares), and link descriptions. The remaining five sheets contain the analyses of the data for this study. For example, Main Data includes only the twelve variables analysed in the present study. Similarly, each of Types & Interactions, Interactions & Time, and Types & Time include data of two variables, and often their cross-tabulations as well. We would like to recommend the researchers who are interested in this dataset utilize the first sheet (i.e. COVID-19 vaccine) for their study or other purposes.

Data are available under the terms of the Creative Commons Zero "No rights reserved" data waiver (CC0 1.0 Public domain dedication).
