# Supplementary material for: An exploratory study of social media users’ engagement with COVID-19 vaccine-related content
Source: F1000Res. 2021 Jun 23;10:236. Originally published 2021 Mar 24. [Version 3] doi: 10.12688/f1000research.51210.3 (PMC8591519; doi:10.12688/f1000research.51210.3)
Supplement: Peer review history

# Peer review: Reviewer response for version 3

**DOI:** 10.5256/f1000research.57966.r97697

**Published:** 2021-11-11

**Authors:** Arriel Benis

**Affiliations:** 1 Faculty of Industrial Engineering and Technology Management, Holon Institute of Technology, Holon, Israel

Thanks to the author for this interesting manuscript.

 The focus on Bangla language and Bangladesh.

 It can be good to add a discussion about the similarities and the differences between the results disclosed herein and other in other countries/areas around the globe. Moreover for the not social media users/specialist it will be good to shortly explain the difference between Facebook, Youtube, Twitter... before explaining why the author choose Facebook ("Data source & search strategy").

Is the work clearly and accurately presented and does it cite the current literature?

Yes

If applicable, is the statistical analysis and its interpretation appropriate?

Yes

Are all the source data underlying the results available to ensure full reproducibility?

No

Is the study design appropriate and is the work technically sound?

Yes

Are the conclusions drawn adequately supported by the results?

Yes

Are sufficient details of methods and analysis provided to allow replication by others?

Yes

Reviewer Expertise:

Health Informatics; Digital Health; Social Media; Artificial Intelligence; Health Communication; Epidemiology;

I confirm that I have read this submission and believe that I have an appropriate level of expertise to confirm that it is of an acceptable scientific standard.

# Peer review: Reviewer response for version 3

**DOI:** 10.5256/f1000research.57966.r88085

**Published:** 2021-06-30

**Authors:** Jayaseelan Rathinaswamy

**Affiliations:** 1 Department of Visual Communication, PSG College of Arts and Science, Coimbatore, Tamil Nadu, 641 014, India

The researchers have given necessary clarification.

Is the work clearly and accurately presented and does it cite the current literature?

Yes

If applicable, is the statistical analysis and its interpretation appropriate?

Yes

Are all the source data underlying the results available to ensure full reproducibility?

Yes

Is the study design appropriate and is the work technically sound?

Yes

Are the conclusions drawn adequately supported by the results?

Partly

Are sufficient details of methods and analysis provided to allow replication by others?

Yes

Reviewer Expertise:

New Media Studies, Developmental Communication are my strong areas of research.

I confirm that I have read this submission and believe that I have an appropriate level of expertise to confirm that it is of an acceptable scientific standard.

# Peer review: Reviewer response for version 2

**DOI:** 10.5256/f1000research.57711.r87721

**Published:** 2021-06-17

**Authors:** Jayaseelan Rathinaswamy

**Affiliations:** 1 Department of Visual Communication, PSG College of Arts and Science, Coimbatore, Tamil Nadu, 641 014, India

Facebook is the most popular social media platform in Bangladesh (94.46%), compared to YouTube (3.31%) and Twitter (0.3%), so we selected it as the source of data for this study. we collected the data from Facebook pages using CrowdTangle: 

- The researchers have mentioned in their study that Facebook is the most popular social medium in Bangladesh. I want to know how they measured it. Have they used any instrument to measure it or did they do any other research on Facebook usage? If they have referred any research paper, then it should be mentioned there. 
    
- CrowdTangle: should be explained clearly.

Most of the Facebook users in Bangladesh who write in the Bangla language use the Bangla alphabet

- The researchers should not use the term “most”. It should clearly be stated what percentage or number of people are using the particular language in social media.

In this study, we followed a bivariate statistical analysis and a simple time-series analysis with one-day intervals. The numbers and percentages are presented in tables, time-series data are presented in graphs.

- The researchers have mentioned about the analysis method but it should be clearer.
- They have mentioned in the paper that the percentage are presented in the table, but have not mentioned in which table. The table number should be mentioned clearly.
- There are no research questions or hypothesis mentioned in the study. It is very important for a research work.  
- Reviews of Literature are very less. More reviews can be added.
- The researchers have used 1st person in some places as 'I' and it should be changed to 3rd person eg: "the researcher/ researchers". (Researchers have not changed this even after mentioning it in the previous report)

Is the work clearly and accurately presented and does it cite the current literature?

Yes

If applicable, is the statistical analysis and its interpretation appropriate?

Yes

Are all the source data underlying the results available to ensure full reproducibility?

Yes

Is the study design appropriate and is the work technically sound?

Yes

Are the conclusions drawn adequately supported by the results?

Partly

Are sufficient details of methods and analysis provided to allow replication by others?

Yes

Reviewer Expertise:

New Media Studies, Developmental Communication are my strong areas of research.

I confirm that I have read this submission and believe that I have an appropriate level of expertise to confirm that it is of an acceptable scientific standard, however I have significant reservations, as outlined above.

# Response

**Published:** 2021-06-19

**Authors:** Md. Sayeed Al-Zaman

**Affiliations:** Jahangirnagar University, Bangladesh

Thanks to the reviewer for further comments on our manuscript. Followings are our responses:

  

1. We did not measure the popularity levels of the social media platforms. Rather, we provided a source from where we borrowed the statistics. Please see the link attached to “ the most popular social media platform in Bangladesh” in the manuscript. As per the policy of F1000 Research, all web citations should be included as links.
2. For “CrowdTangle”, see point 1. Also, we defined CrowdTangle as “a public insights tool owned and operated by Facebook” in the manuscript, which is an ideal and referred description of this tool. For more, see https://help.crowdtangle.com/en/articles/3192685-citing-crowdtangle-data.
3. We excluded the word “most”. However, a specific percentage is absent regarding the statement, and measurement of the entire Facebook users is not possible as well. For that reason, we had to rely on our general observations.
4. Regarding the analysis, we do not think the method needs further clarification because it clearly mentions the two techniques we devised in this study. Bivariate analysis is simply the cross-tabulation between two variables and time series analysis is the distribution of a variable throughout a specific period. Detailing such a primary-level explanation would make the section verbose.
5. Since all tables are cross-tabulations produced by bivariate analysis, it would not require mentioning the table number in the method section. Rather, we clearly mention the number in the result section while interpreting our data.
6. F1000 Research is a medicine-related journal, and such journals unlike the social sciences and humanities journals emphasize results over literature review. Research papers in similar journals should be as succinct as possible, though we provide the reviews of necessary studies for both the background and conceptual framework. Also, the guideline of the journal clearly states that the four required sections should be: Introduction, Methods, Result, Conclusions/Discussion. Therefore, we decided not to include a dedicated Literature Review section for a detailed literature review. Lastly, since this is an exploratory study, it is obvious that not many studies have not been conducted in this area so far.
7. One “I” was remained in the manuscript mistakenly and we have replaced it with “we.” Thanks for the suggestion. As mentioned in our previous response to the reviewer’s comments, using “we” in scholarly manuscripts is standard practice.

We have now addressed the reviewer’s concerns properly and make the necessary changes (see Track Change) in the manuscript.

# Peer review: Reviewer response for version 1

**DOI:** 10.5256/f1000research.54353.r86107

**Published:** 2021-06-09

**Authors:** Jayaseelan Rathinaswamy

**Affiliations:** 1 Department of Visual Communication, PSG College of Arts and Science, Coimbatore, Tamil Nadu, 641 014, India

The results of the study should be more focused towards implications, which is missing. The conclusion gives positive and negative aspects of Facebook on COVID-19 and but doesn't give a strong conclusion. 

 The Researcher/s have used time frame analysis but not mentioned which type of approach they have adopted. 

 The researcher used 1st person in some places as 'I' and it should be changed as 3rd person eg: "the researcher/ researchers".

Is the work clearly and accurately presented and does it cite the current literature?

Yes

If applicable, is the statistical analysis and its interpretation appropriate?

Yes

Are all the source data underlying the results available to ensure full reproducibility?

Yes

Is the study design appropriate and is the work technically sound?

Yes

Are the conclusions drawn adequately supported by the results?

Partly

Are sufficient details of methods and analysis provided to allow replication by others?

Yes

Reviewer Expertise:

New Media Studies, Developmental Communication are my strong areas of research.

I confirm that I have read this submission and believe that I have an appropriate level of expertise to confirm that it is of an acceptable scientific standard.

# Response

**Published:** 2021-06-10

**Authors:** Md. Sayeed Al-Zaman

**Affiliations:** Jahangirnagar University, Bangladesh

Many thanks to the reviewer for such valuable comments on this manuscript. The following changes have been made using Track Change according to the suggestions:

  

1. Thanks for pointing out the apparent limitation regarding the result interpretation. However, a glimpse of the intellectual implication of the results has already been included at the end of the conclusion (please, see from “ *Apart from these limitations, this study has strengths in a few areas…*” to “ *Finally, this research dealt with a larger dataset than the previous studies on the COVID-19 vaccine…*”). Also, the last sentence would inform the readers that this is an exploratory study, and such studies might have limitations from different aspects (e.g., reaching some certain and concrete conclusions), but it can guide future studies in this area. From this perspective, the inability to forecast very concise practical and policy-related significance could be difficult for this study.
2. A one-sentence clarification regarding the data analysis has now been added to the methodology.
3. The use of “I” is now replaced with “we”, which is an ideal practice in the scholarly arena.
